# Supplementary material for: Synthesis and Structure–Activity Relationship of Salvinal Derivatives as Potent Microtubule Inhibitors
Source: Int J Mol Sci. 2023 Mar 28;24(7):6386. doi: 10.3390/ijms24076386 (PMC10093915; doi:10.3390/ijms24076386)
Supplement: Supplementary file 1 [file ijms-24-06386-s001.zip › ijms-2258401-supplementary.pdf]

SUPPLEMENTARY MATERIAL

Synthesis and Structure-Activity Relationship of Salvinal Derivatives as Potent Microtubule Inhibitors

Chi-I Chang [1,†], Cheng-Chih Hsieh [2,3,†], Yung-Shung Wein [4], Ching-Chuan Kuo [5,6], Chi-Yen Chang [7], Jrhau Lung [8], Jong-Yuh Cherng [9], Po-Chen Chu [10], Jang-Yang Chang [5,11, 12,*], Yueh-Hsiung Kuo [13,14,15,*]

[1] Department of Biological Science and Technology, National Pingtung University of Science and Technology, Pingtung 912, Taiwan
[2] Department of pharmacy, Kaohsiung Veterans General Hospital, Kaohsiung 813, Taiwan
[3] School of Pharmacy and Institute of Pharmacy, National Defense Medical Center, Taipei 114, Taiwan
[4] Department of Chemistry, National Taiwan University, Taipei 114, Taiwan
[5] Institute of Biotechnology and Pharmaceutical Research, National Health Research Institutes, Miaoli 350, Taiwan
[6] Graduate Institute of Biomedical Sciences, China Medical University, Taichung 404, Taiwan; Road, Tainan 704, Taiwan
[7] National Institute of Cancer Research, National Health Research Institutes, Miaoli 350, Taiwan
[8] Department of Medical Research and Development, Chiayi Chang Gung Memorial Hospital, Chiayi Branch, Chiayi 613, Taiwan
[9] Department of Chemistry and Biochemistry, National Chung Cheng University, Chiayi 613, Taiwan
[10] Department of Cosmeceutics and Graduate Institute of Cosmeceutic, China Medical University, Taichung 404, Taiwan
[11] Taipei Cancer Center, Taipei Medical University Hospital, Taipei 110, Taiwan
[12] TMU Research Center of Cancer Translational Medicine, Taipei Medical University, Taipei 110, Taiwan
[13] Department of Chinese Pharmaceutical Sciences and Chinese Medicine Resources, College of Pharmacy, China Medical University, Taichung 404, Taiwan
[14] Department of Biotechnology, Asia University, Taichung 413, Taiwan
[15] Chinese Medicine Research Center, China Medical University, Taichung 404, Taiwan

CONTENT

Figure S1. ^{1}H-NMR spectrum of compound **4** in $CDCl_3$
Figure S2. ^{13}C-NMR spectrum and DEPT of compound **4** in $CDCl_3$
Figure S3. IR spectrum of compound **4**
Figure S4. Mass spectrum of compound **4**
Figure S5. ^{1}H-NMR spectrum of compound **19** in $CDCl_3$
Figure S6. ^{13}C-NMR spectrum and DEPT of compound **19** in $CDCl_3$
Figure S7. IR spectrum of compound **19**
Figure S8. Mass spectrum of compound **19**
Figure S9. ^{1}H-NMR spectrum of compound **20** in $CDCl_3$
Figure S10. ^{13}C-NMR spectrum and DEPT of compound **20** in $CDCl_3$
Figure S11. IR spectrum of compound **20**
Figure S12. Mass spectrum of compound **20**
Figure S13. ^{1}H-NMR spectrum of compound **25** in $CDCl_3$
Figure S14. ^{13}C-NMR spectrum and DEPT of compound **25** in $CDCl_3$
Figure S15. IR spectrum of compound **25**
Figure S16. Mass spectrum of compound **25**

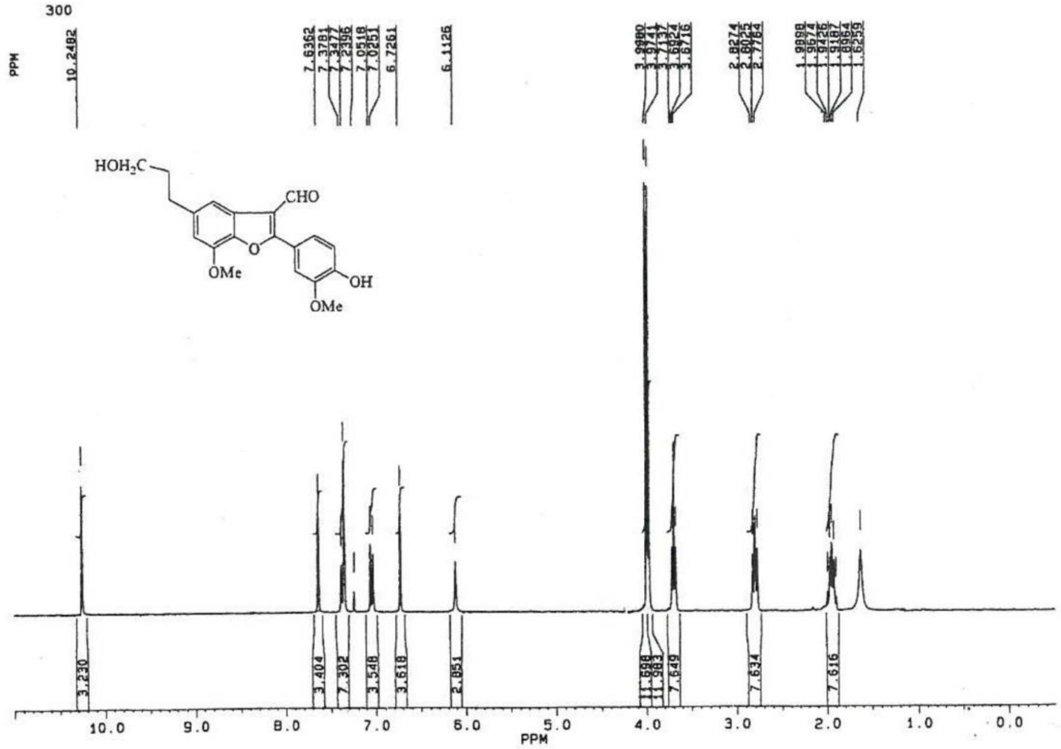

Figure S1. ^{1}H-NMR spectrum of compound **4** in $CDCl_3$

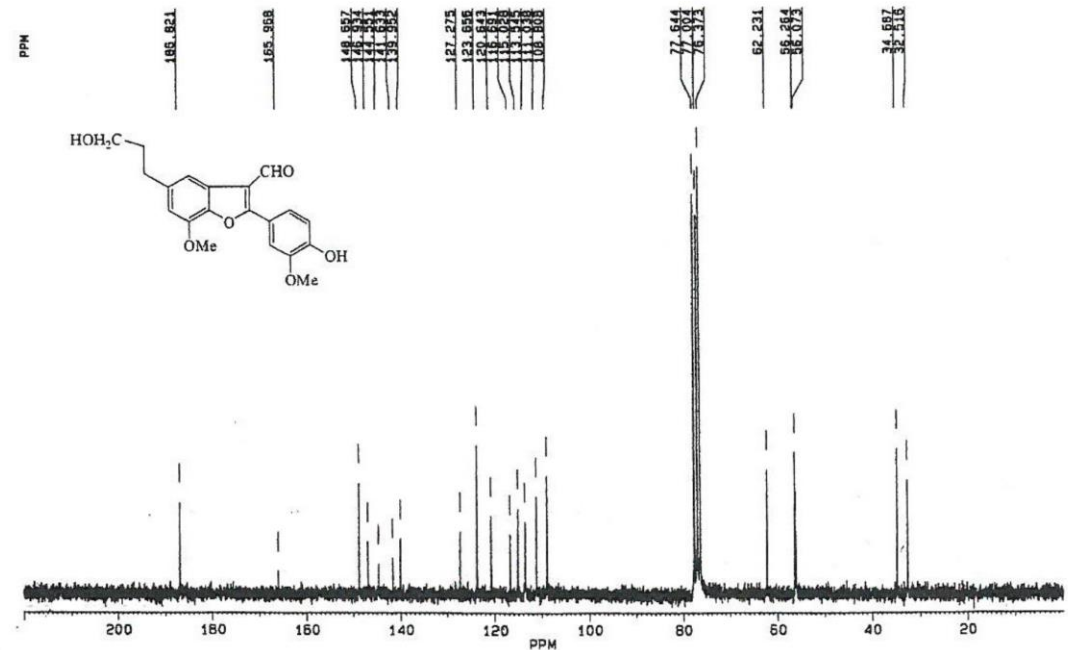

Figure S2. ^{13}C-NMR spectrum and DEPT of compound **4** in $CDCl_3$

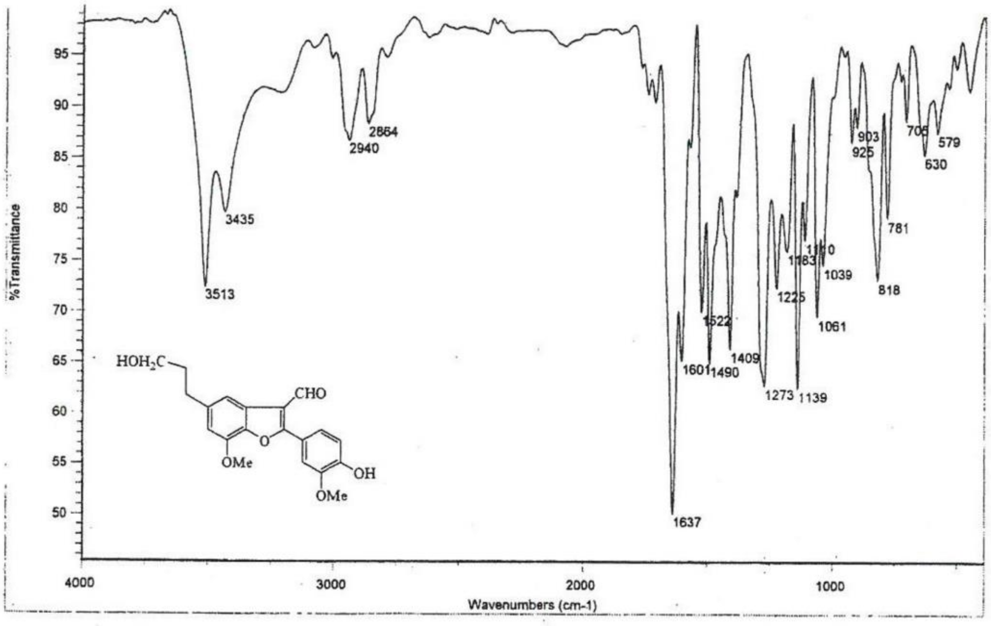

Figure S3. IR spectrum of compound **4**

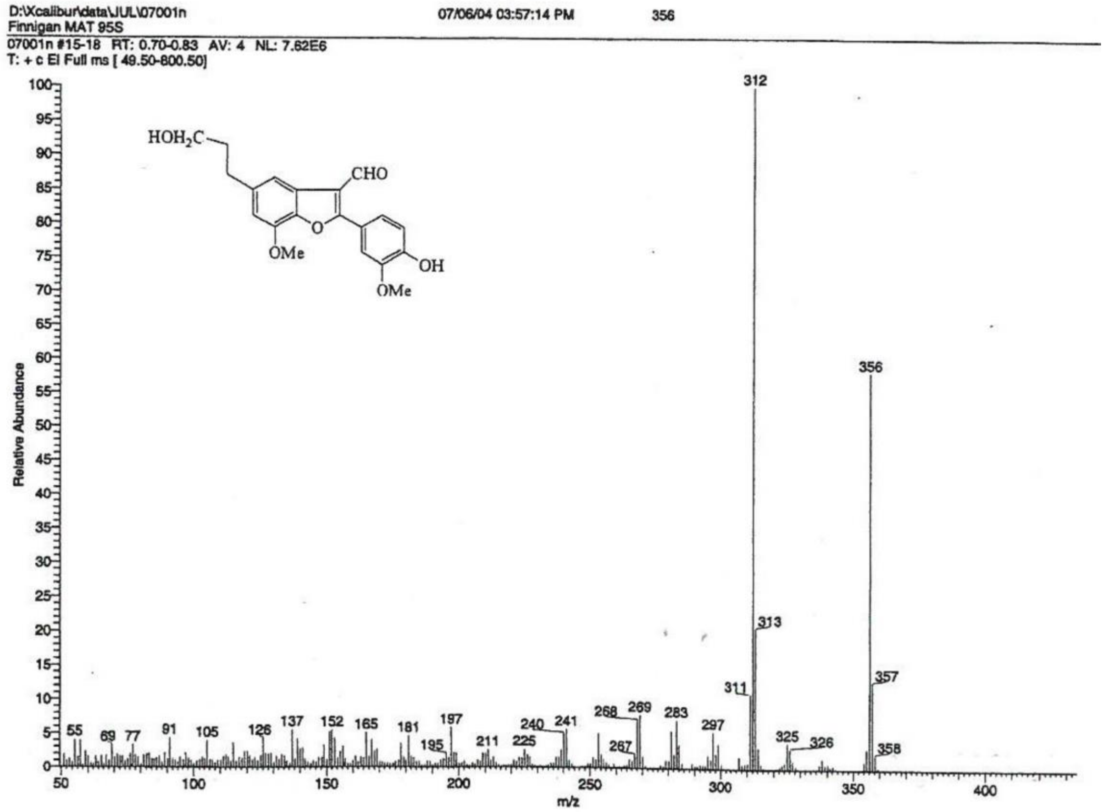

Figure S4. Mass spectrum of compound **4**

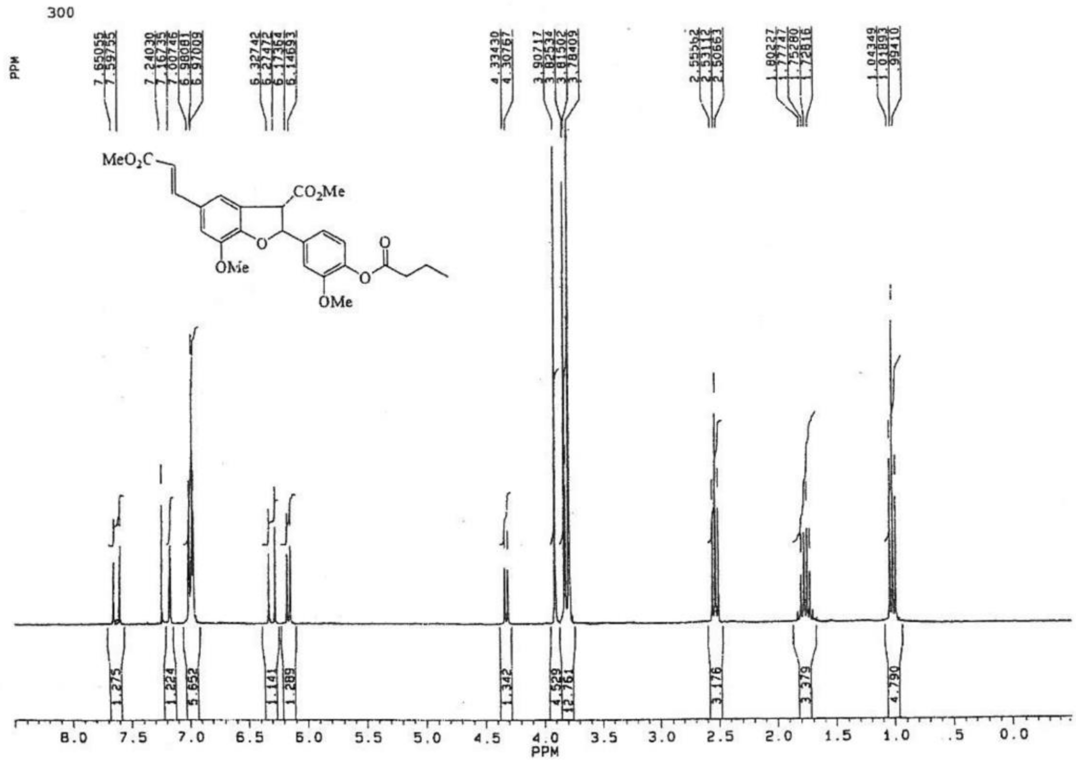

Figure S5. ^{1}H-NMR spectrum of compound **19** in $CDCl_3$

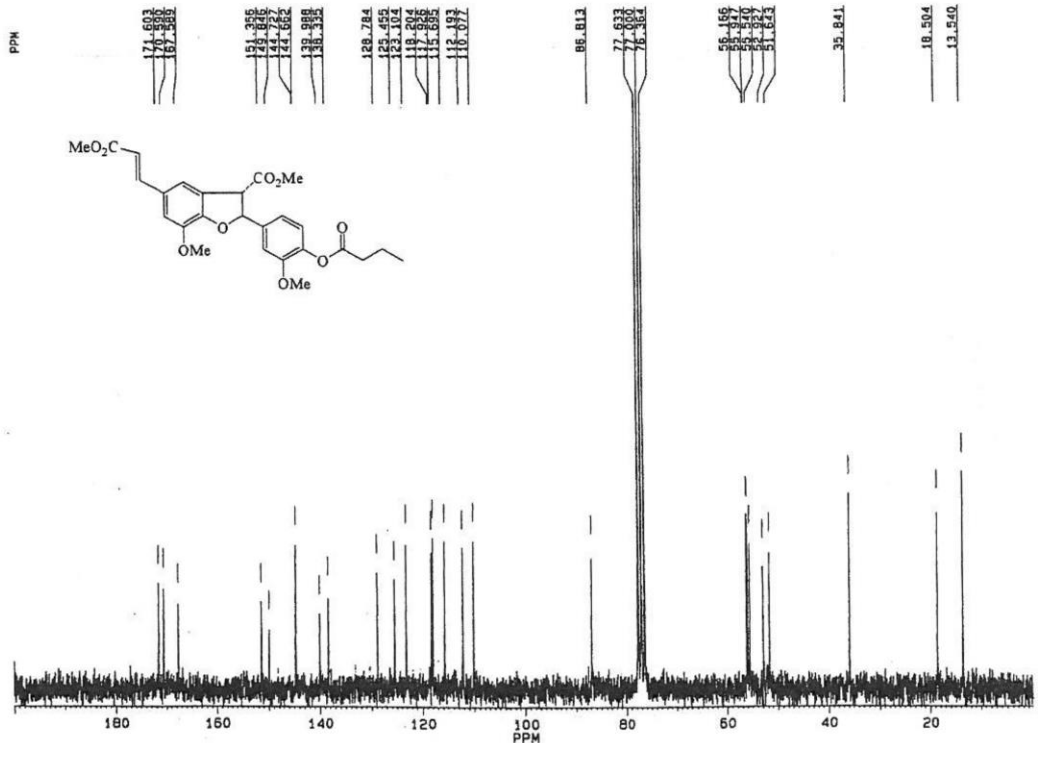

Figure S6. ^{13}C-NMR spectrum and DEPT of compound **19** in $CDCl_3$

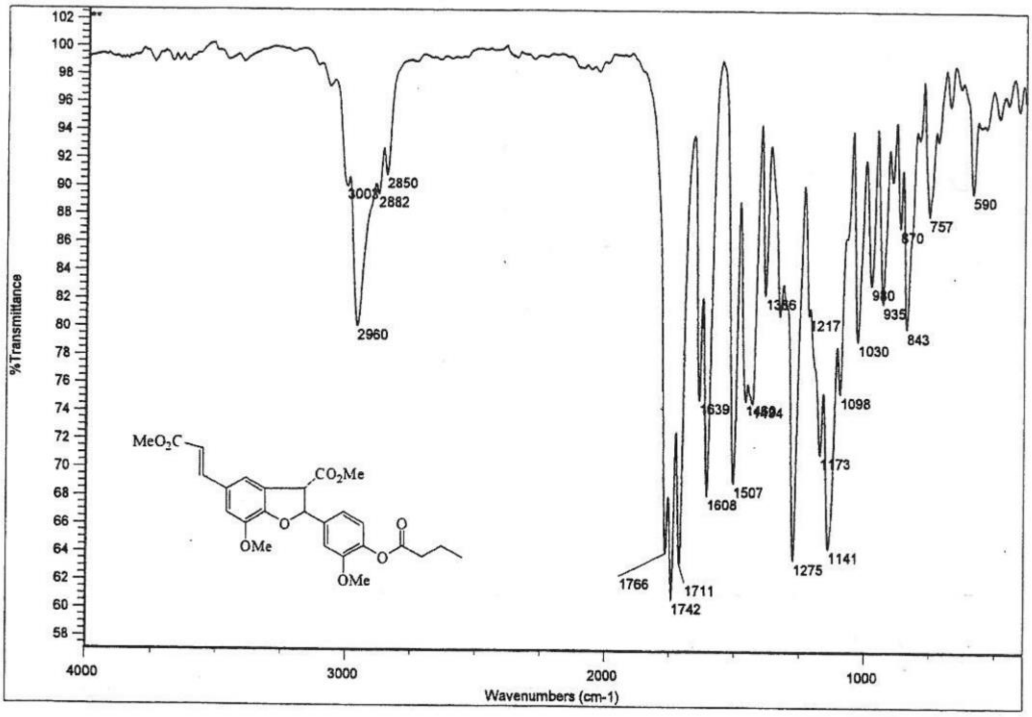

Figure S7. IR spectrum of compound **19**

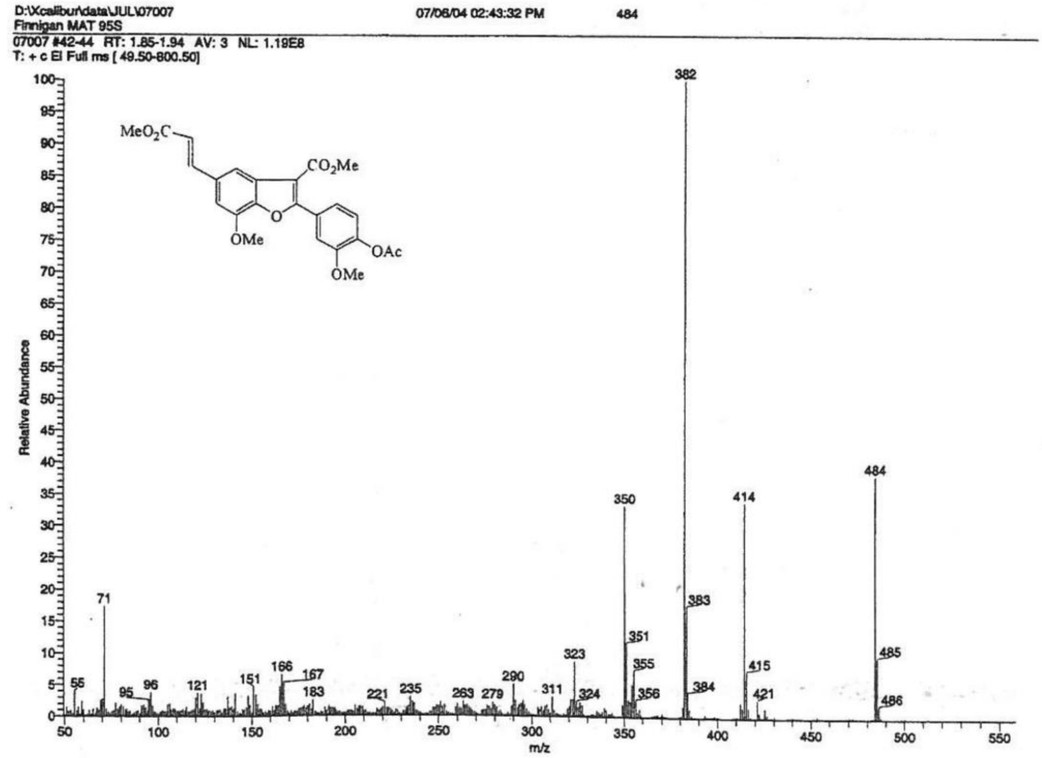

Figure S8. Mass spectrum of compound **19**

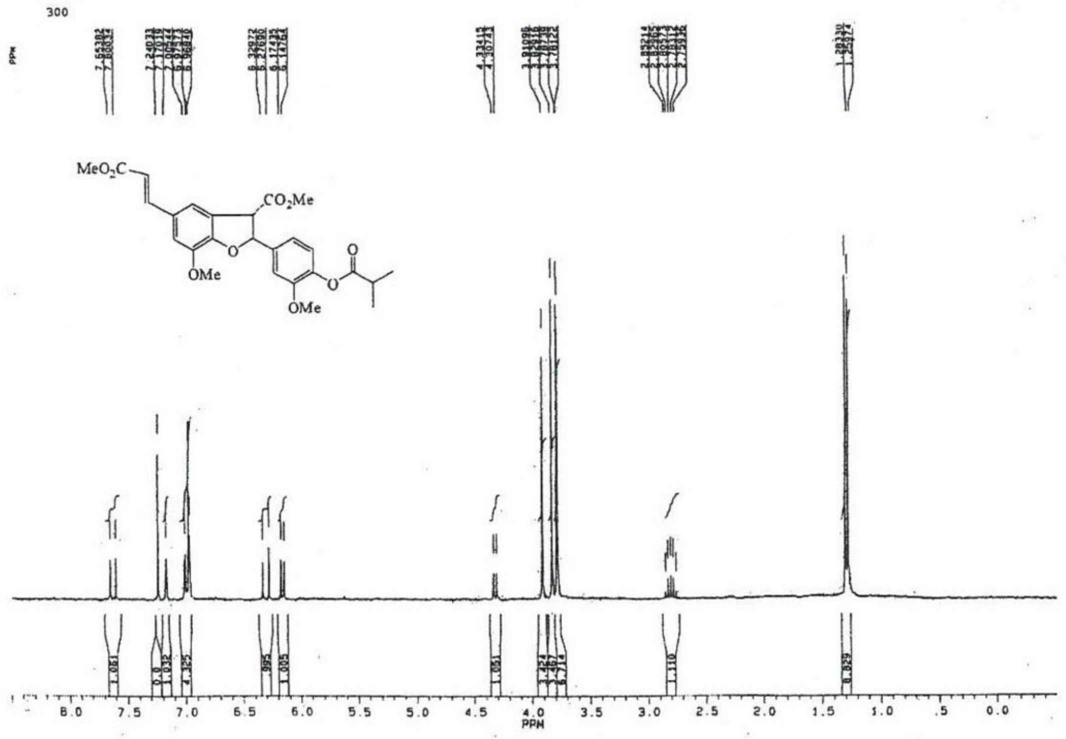

Figure S9. ^{1}H-NMR spectrum of compound **20** in $CDCl_3$

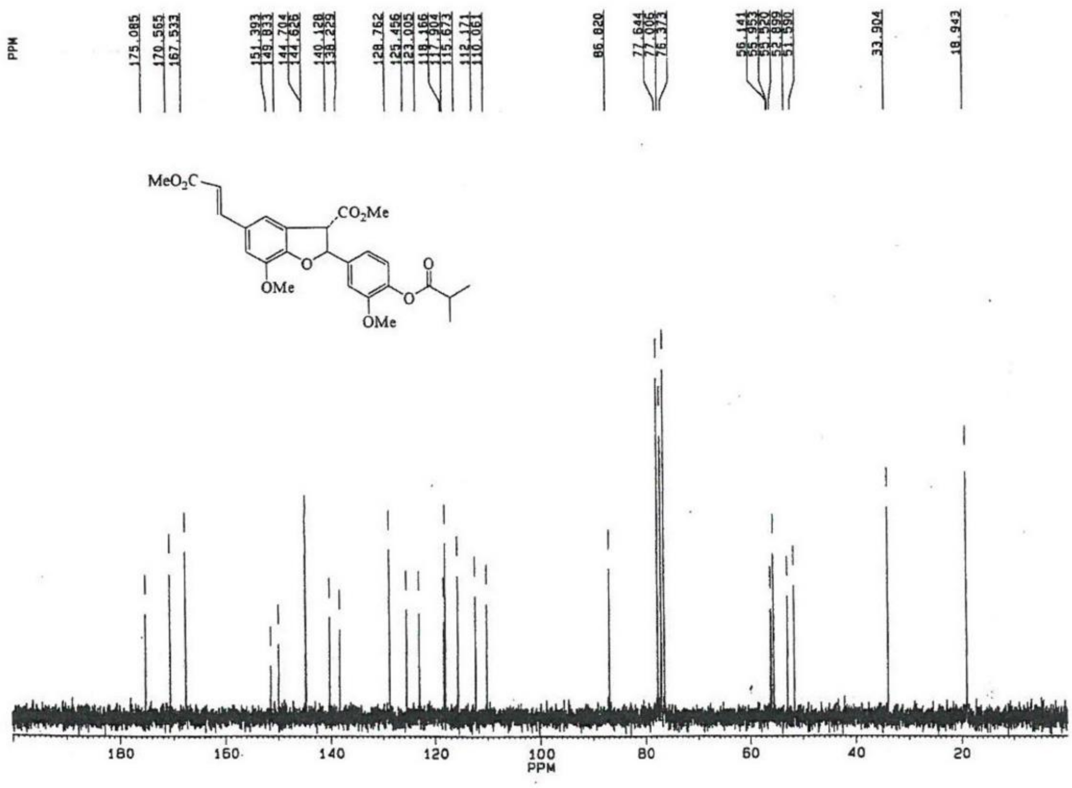

Figure S10. ^{13}C-NMR spectrum and DEPT of compound **20** in $CDCl_3$

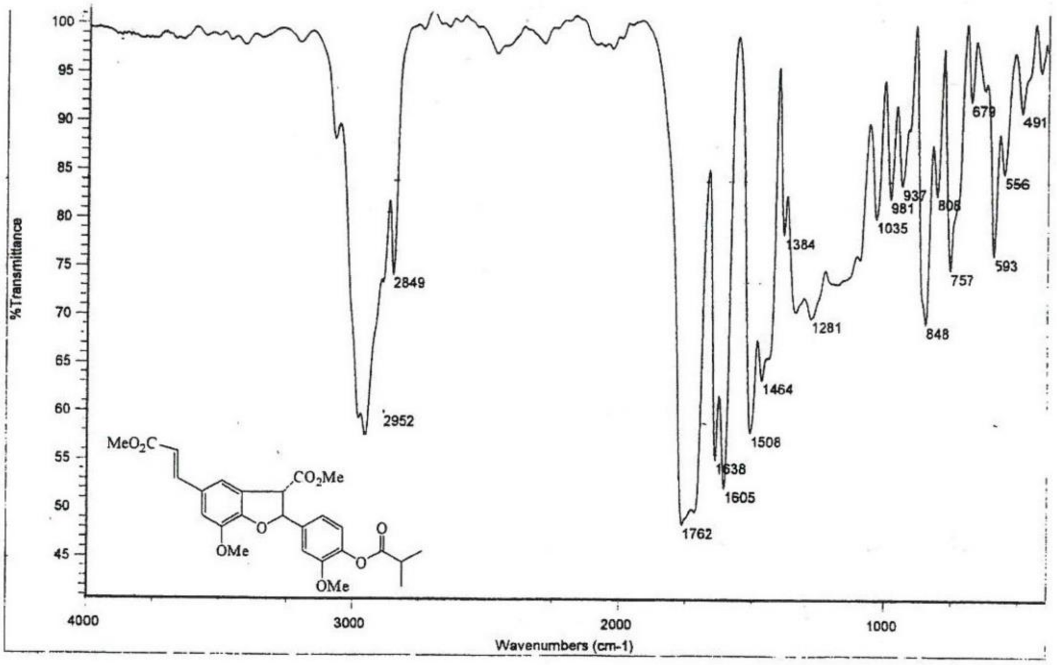

Figure S11. IR spectrum of compound **20**

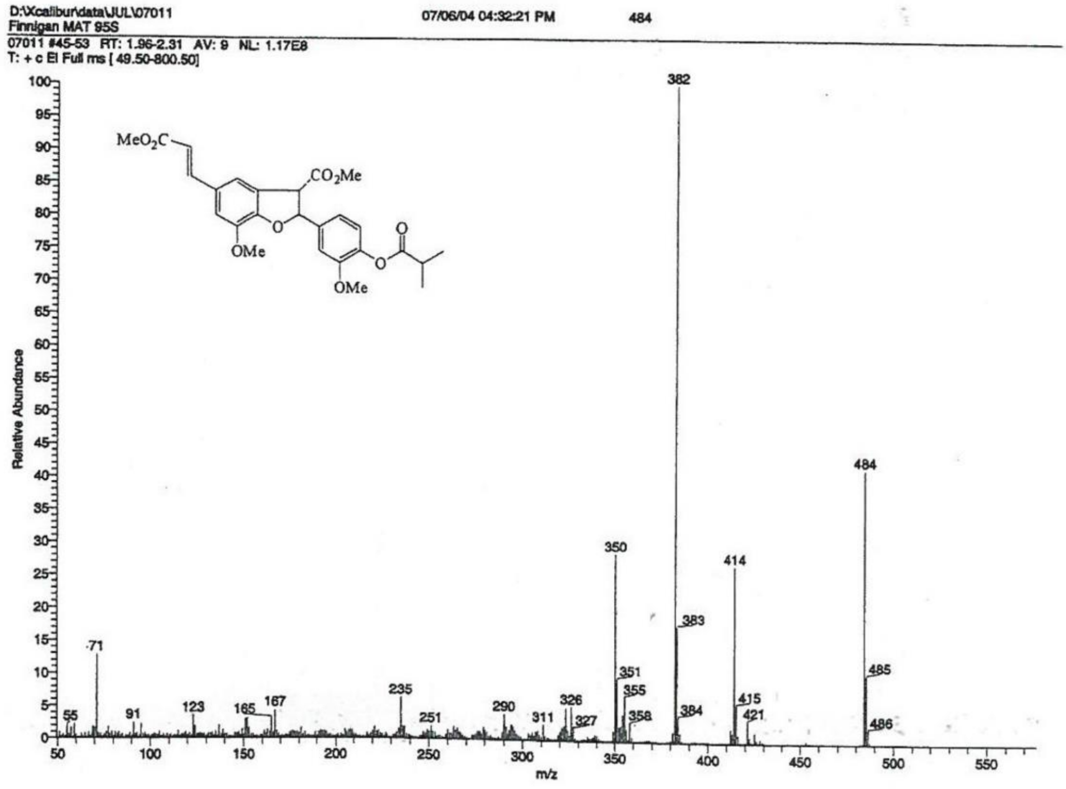

Figure S12. Mass spectrum of compound **20**

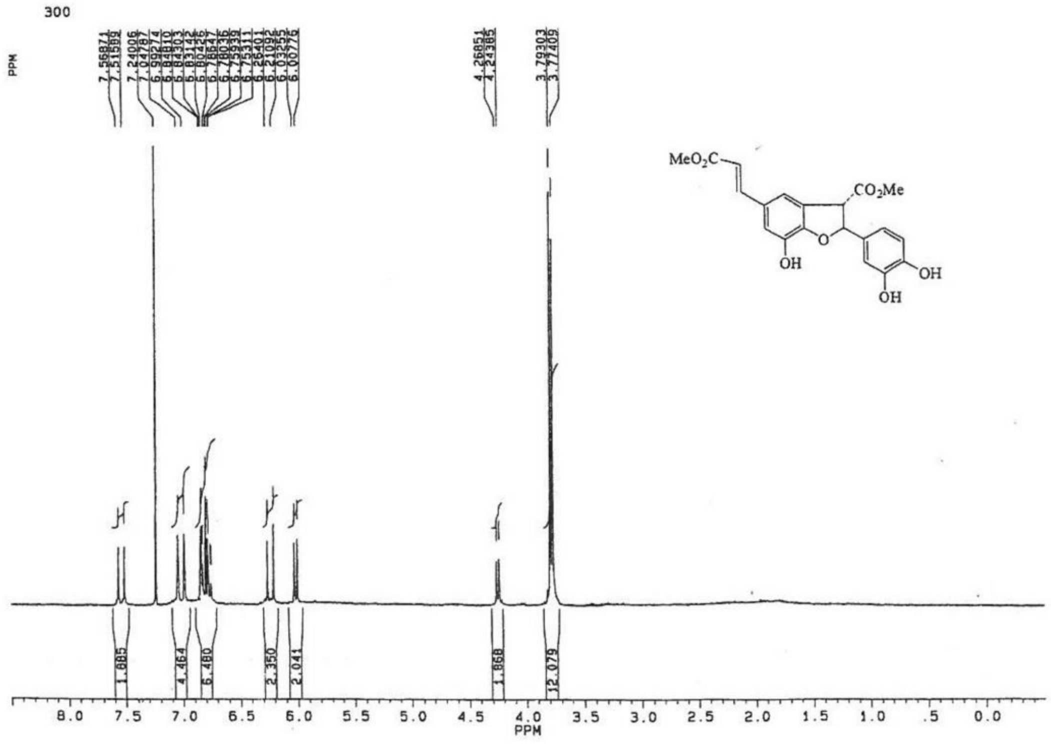

Figure S13. ^{1}H-NMR spectrum of compound **25** in $CDCl_3$

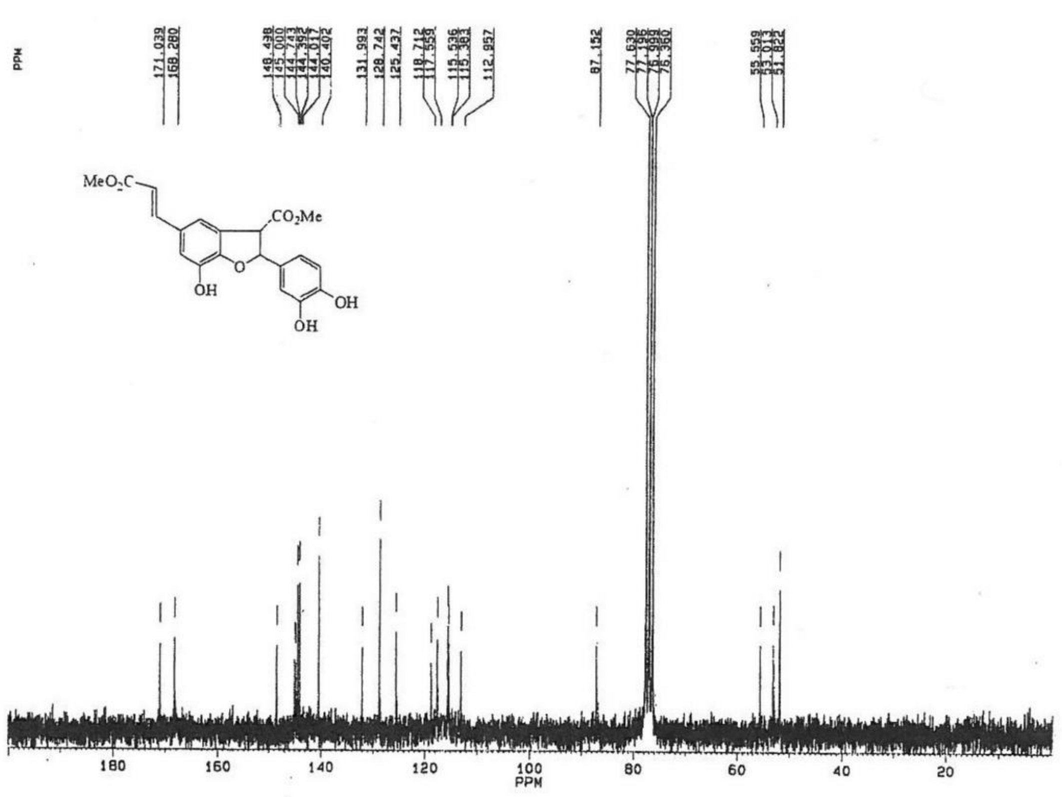

Figure S14. ^{13}C-NMR spectrum and DEPT of compound **25** in $CDCl_3$

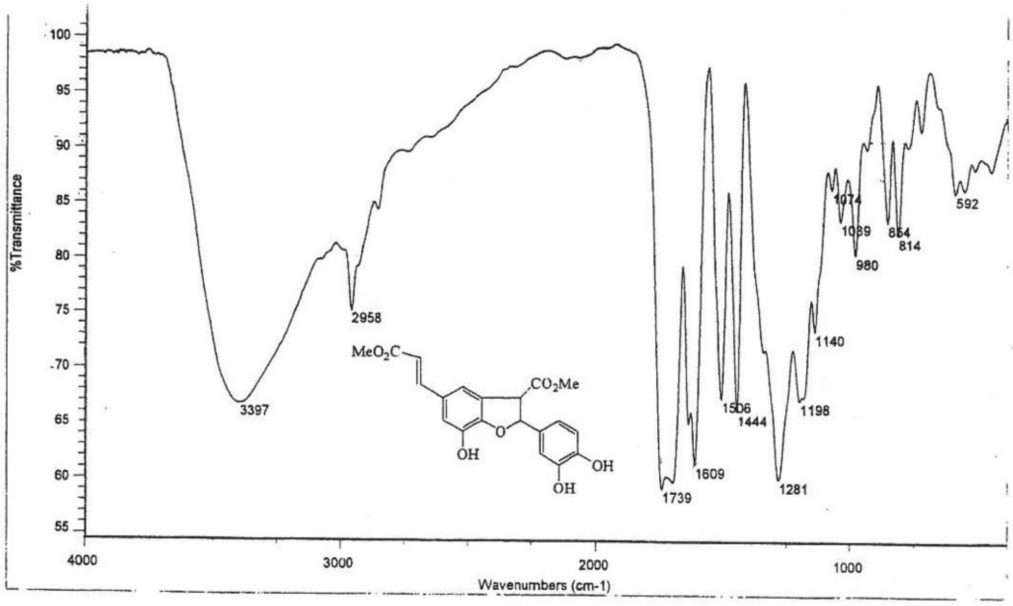

Figure S15. IR spectrum of compound **25**

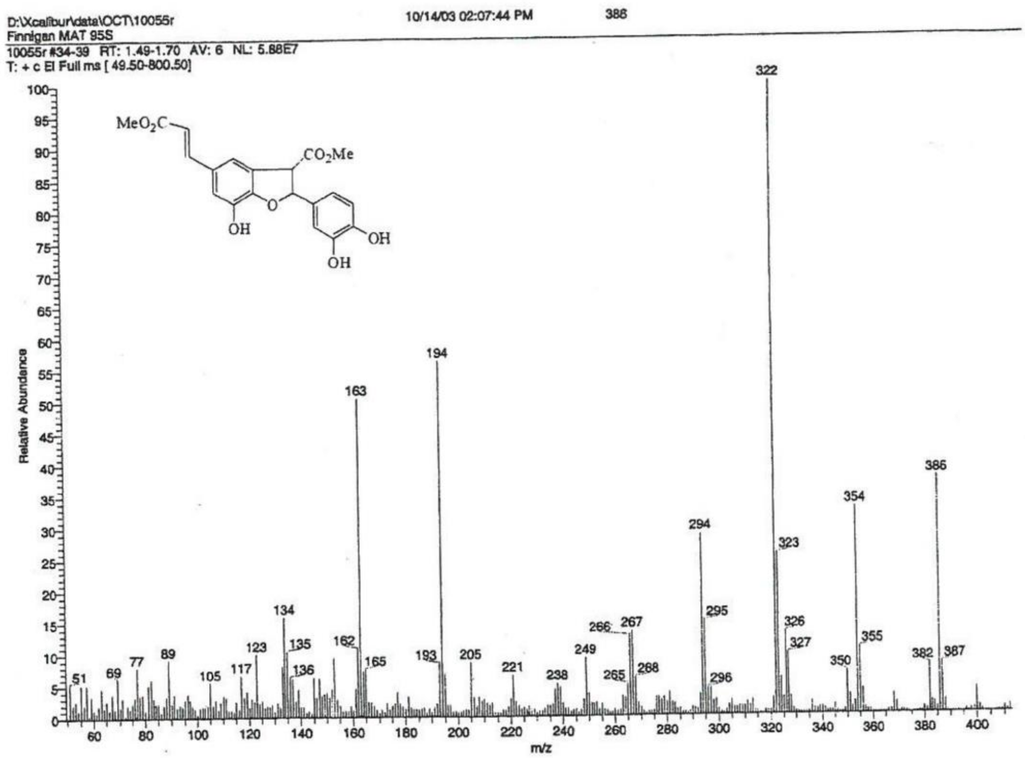

Figure S16. Mass spectrum of compound **25**
